# PCL/POSS Nanocomposites: Effect of POSS Derivative and Preparation Method on Morphology and Properties

**DOI:** 10.3390/polym11010033

**Published:** 2018-12-26

**Authors:** Mónica Cobos, Johnny R. Ramos, Dailyn J. Guzmán, M. Dolores Fernández, M. Jesús Fernández

**Affiliations:** Department of Polymer Science and Technology, Faculty of Chemistry, University of the Basque Country (UPV/EHU), P° Manuel Lardizábal 3, 20018 San Sebastián, Spain; monica.cobos@ehu.es (M.C.); johnram@hotmail.com (J.R.R.); daylyngm@yahoo.es (D.J.G.); mariadolores.fernandez@ehu.es (M.D.F.)

**Keywords:** poly(ε-caprolactone) nanocomposite, POSS nanoparticles, dispersion, morphology, thermal properties, mechanical properties, surface properties

## Abstract

The incorporation of polyhedral oligomeric silsesquioxanes (POSS) molecules as nanoparticles into polymers can provide improved physico-chemical properties. The enhancement depends on the extent of dispersion of the nanofiller, which is determined by the compatibility with the polymer that is by the POSS type, and the processing method. In this study, poly(ε-caprolactone)/POSS derivatives nanocomposites (PCL/POSS) were obtained via solution-casting and melt compounding. Two amino-derivatives containing different alkyl substituents, and ditelechelic POSS-containing hybrid PCL masterbatch were used as nanofillers. The effect of preparation method, POSS content and type on the morphology, thermal, mechanical, and surface properties of nanocomposites were studied. Morphological analysis evidenced the formation of POSS crystalline aggregates, self-assembled POSS molecules of submicrometer size dispersed in the polymer matrix. The best dispersion was achieved using the ditelechelic POSS-containing hybrid PCL masterbatch, and comparing the two amino-POSS derivatives, the one with longer alkyl chain of substituents exhibited better degree of dispersion independent of preparation method. DSC analysis showed the role of POSS derivatives as nucleating agents for PCL. The incorporation of POSS derivatives into the PCL matrix improved thermal stability. The preparation method, POSS type and content had influence on mechanical properties of nanocomposites. POSS nanoparticles enhanced the surface hydrophobicity of PCL.

## 1. Introduction

In recent years, the growing problem of waste disposal has sparked a great interest in biodegradable polymers [1,2,3,4]. Polycaprolactone (PCL) is one of the most attractive biodegradable synthetic polymers due to its commercial availability, biodegradability, and compatibility with different forms of waste disposal and excellent physico-mechanical properties [5]. PCL, a semicrystalline linear aliphatic thermoplastic polyester synthetized by the ring-opening polymerization of caprolactone, exhibits good flexibility, non-toxicity, hydrophobicity, low melting point (58–60 °C) and glass-transition temperature (−60 °C), ease of processing, and compatibility with a range of other polymers [6,7,8]. PCL has found extensive applications as commodity and biomedical materials, and for agricultural uses [7,9,10,11], as a substitute material for non-biodegradable polymers. However, modification is highly necessary when it is applied to different requirements. Using nanoparticles significantly improved the properties of PCL [12,13].

Among the variety of nanoscale fillers, polyhedral oligomeric silsesquioxanes (POSS) nanoparticles are considered as one of the most interesting nanofillers that have been used in the preparation of nanocomposites. POSS are a new class of organic/inorganic hybrid materials exhibiting a specific three dimensional cage structure made up of Si–O, with size from 1.5 to 3 nm in diameter. The tetravalent Si atoms of POSS are bound to organic groups, one or more of which may contain reactive functional groups. POSS additives can be incorporated into organic polymers via copolymerization, grafting, or blending [14,15,16,17]. Many studies have demonstrated the enhancement in the properties of polymers (thermal and oxidation resistance, mechanical properties, surface hardening, as well as reduced flammability) by incorporating POSS. The dispersion and distribution of nanoparticles in the polymer as well as the interfacial adhesion are key factors for improving the polymer properties. A nanometric dispersion and good distribution is necessary. The morphology depends on the processing method, the chemical structure and concentration of the nanoparticles [18,19]. To achieve the best morphology several methodologies can be used, functional groups can be introduced on the POSS molecules, polymer chains can be grafted on the POSS surface, as well as the optimization of the preparation method.

In previous works, different POSS derivatives have been incorporated into PCL [20,21,22,23,24,25]. Goffin et al. [20] studied the addition of aminopropylheptakis(isobutyl)-POSS and POSS-*g*-PCL nanohybrid by melt blending into PCL. Individualized POSS nanoparticles dispersed in the nanocomposites were only found when POSS-*g*-PCL nanohybrid was used as a masterbatch. Nanocomposites with enhanced crystallinity and thermo-mechanical properties were obtained. Miltner et al. [21] reported the preparation of POSS nanocomposites based on PCL by in situ polymerization of ε-caprolactone in the presence of aminopropylheptakis(isobutyl)-POSS or by melt mixing techniques, and using the nanohybrid prepared by in situ polymerization as masterbatch for melt mixing with PCL. They found amino-POSS agglomerates arranged in crystalline structure in the nanocomposite prepared by melt mixing, whereas enhanced dispersion quality was observed in the case of the nanocomposite prepared by in situ polymerization. As for the nanocomposite prepared by the masterbatch approach, they found that a higher grafted chain length was more efficient in improving the compatibility between POSS and the PCL matrix. Pan et al. [22] studied the morphology and crystallization of PCL/octaisobutyl-POSS nanocomposites prepared via solution casting method. Aggregates of submicron sized POSS particles were found and enhanced crystallization of PCL. Guan and Qiu [23] studied the crystallization, morphology, and dynamic mechanical properties of PCL in the PCL/octavinyl-POSS nanocomposites obtained by solution blending. They observed fine dispersion, enhanced overall isothermal melt crystallization rates of PCL, and improved storage modulus of the nanocomposites. Miltner et al. [24] investigated the influence of addition of aminopropylheptakis(isobutyl)-POSS into PCL via melt mixing on the thermal properties. A fraction of aggregated POSS structures were observed that did not exerted nucleating effect on PCL crystallization. Lee and Chang [25] reported the effect of trisilanolphenyl-POSS on the thermal and mechanical properties of PCL/POSS nanocomposites prepared by solution mixing. They found that POSS molecules were able to crystallize in the PCL matrix, a decreased degree of crystallinity of PCL, and increased tensile properties.

The purpose of the present study is to investigate the effects of POSS type, POSS concentration, and preparation method (solution-casting and melt compounding) on the morphology, thermal, mechanical, and surface properties of the PCL/POSS nanocomposites. Two amino-derivative POSS containing different alkyl group, aminopropylheptaisobutyl-POSS (APIBPOSS) and aminopropylheptaisooctyl-POSS (APIOPOSS), and ditelechelic POSS-containing hybrid poly(ε-caprolactone) masterbatch, propyl-heptaisooctyl-POSS-*g*-PCL-*g*-propyl-heptaisooctyl-POSS (PIOPOSS–PCL–PIOPOSS), were used as fillers to prepare PCL/POSS nanocomposites. X-ray diffraction (XRD) and transmission electron microscopy (TEM) were used to examine the state of dispersion of POSS nanoparticles in the nanocomposites. Thermal properties were determined using differential scanning calorimetry (DSC) and thermogravimetric analysis (TGA). The mechanical properties were investigated through tensile tests. Surface properties of nanocomposites were studied by contact angle measurements.

Aminopropyl-heptaisobutyl-POSS is the only amino-POSS derivative used in the so far reported studies focused on PCL/POSS nanocomposites, and neither the effect of preparation method (solution casting and melt mixing), nor the effect of POSS substituent group have been studied [20,21,22,23,24,25]. The compatibility with the polymer and the dispersion state depends on the nature of the organic groups (R) of POSS molecules, and hence the properties of the new material. Thus, two amine-functionalized POSS were used in this study to investigate the effect of the alkyl chain length of the nonreactive organic substituents attached to the corner silicon atoms on morphology, thermal, and mechanical properties, as well as surface hydrophobicity of PCL. In addition, the masterbatch POSS-PCL-POSS was used to prepared PCL nanocomposites to make a comparison with ungrafted POSS nanoparticles.

## 2. Materials and Methods

### 2.1. Materials

**Figure 1:**
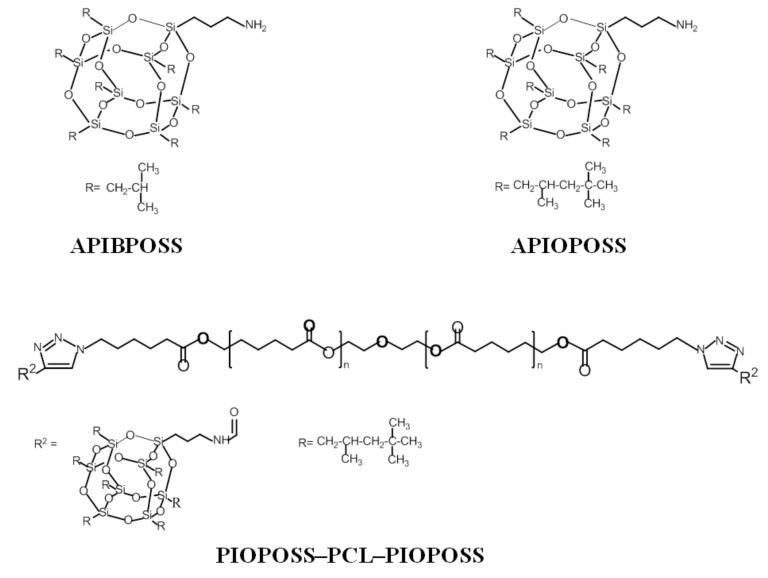
The chemical structure of polyhedral oligomeric silsesquioxanes (POSS) types used in this work.

Poly(ε-caprolactone) (PCL) (number-average molar mass = 45,000 g/mol), was supplied by Sigma-Aldrich (Munich, Germany). APIBPOSS (C_31_H_71_NO_12_Si_8_, Fw = 874.58), a white crystalline powder, and APIOPOSS (C_59_H_127_NO_12_Si_8_, Fw = 1267.32), a pale yellow viscous liquid, were purchased from Hybrid plastics (Hattiesburg, MS, USA). PIOPOSS-PCL-PIOPOSS hybrid was synthesized by grafting APIOPOSS to PCL chains using “click chemistry” as described elsewhere [26]. The alkyne-functionalized POSS (*N*-(3-(heptaisooctyl POSS) propyl) propiolamide) and bis-azide end-functionalized PCL were used for the click chemistry approach. Figure 1 shows the chemical structure of each POSS. Trichloromethane (CHCl_3_) was obtained from Scharlau (Barcelona, Spain).

### 2.2. Preparation of PCL/POSS Nanocomposites

PCL/APIBPOSS, PCL/APIOPOSS, and PCL/PIOPOSS-PCL-PIOPOSS masterbatch nanocomposite films were prepared by solution blending using trichloromethane as solvent. PCL and the appropriate amounts of POSS were dissolved separately. These two solutions were mixed together and stirred with sonication for 1 h, then cast into glass plates, and after solvent evaporation at room temperature the films were dried in a vacuum oven at 60 °C for 3 days. The nanocomposites were named PCL/APIBPOSS-x-S, PCL/APIOPOSS-x-S, and PCL/PIOPOSS-PCL-PIOPOSS-x-S, where x denotes the weight percentage of POSS derivative.

Melt compounded PCL/APIBPOSS and PCL/APIOPOSS nanocomposites at 2, 5, and 10 wt.% of POSS concentration were prepared in a Minilab II, Haake Rheomix CTW5 mini twin-screw extruder (Waltham, MA, USA) (90 °C, 10 min, 50 rpm). For characterization purposes, the extruded mixture was compression-molded at 60 °C and 300 bars of pressure for 5 min. The nanocomposites were named PCL/APIBPOSS-x-M and PCL/APIOPOSS-x-M where x denotes the weight percentage of POSS derivative.

### 2.3. Characterization of PCL/POSS Nanocomposites

XRD patterns were recorded on a Bruker D8 Advance X-ray diffractometer (Karlsruhe, Germany) with a graphite monochromator, Cu Kα generator (λ = 0.154 nm), and operating at 40 kV/30 mA.

TEM micrographs were obtained using a Philips Tecnai G2 20 TWIN TEM (Eindhoven, the Netherlands) at 200 kV accelerated voltage. The samples were cryo-ultramicrotomed using a Leica EM UC6 ultramicrotome apparatus and placed onto Cu grids.

Molecular weight of the PCL matrix (before and after nanocomposites preparation) was determined by gel permeation chromatography (GPC) analysis, using a Waters 2410 HPLC instrument (Milford, MA, USA) equipped with Styragel columns calibrated with polystyrene standards, with tetrahydrofuran as eluent with the flow rate of 1 mL min^−1^.

DSC analyses were carried out using a TA instruments TA-DSC Q2000 (New Castle, DE, USA) under nitrogen atmosphere at a heating rate of 10 °C min^−1^. The samples, about 6 mg, were heated from −100 to 100 °C and held in the molten state for 5 min (to erase the thermal history), then cooled to −60 °C at 10 °C min^−1^ and held 5 min, and again heated to 100 °C at 10 °C min^−1^.

Thermogravimetric analyses were performed on a TA instruments His Res TG-Q-500 instrument (New Castle, DE, USA) under nitrogen or air atmosphere from 50 to 800 °C at a heating rate of 10 °C min^−1^. The thermal degradation temperature was taken as the onset temperature at which 5% of weight loss occurs.

Tensile tests were carried out with a Universal mechanical testing machine Instron model 5569 (Norwood, MA, USA) according to ASTM D 638, at room temperature, gauge length of 35 mm and speed of 5 mm min^−1^. Testing was carried out on at least five identical samples of each composition and the average values were reported.

Static contact angles were measured by using the sessile drop technique with a digital goniometer (Filderstadt, Germany) equipped with a dispensing needle (Dataphysics Contact Angle System OCA), using deionized water as liquid. At least 10 measurements were taken at different locations of the each sample surface and average values of static contact angles were reported.

## 3. Results and Discussion

### 3.1. Effect of POSS Type and Processing Method on Morphology and Dispersion of POSS in PCL Matrix

**Figure 2:**
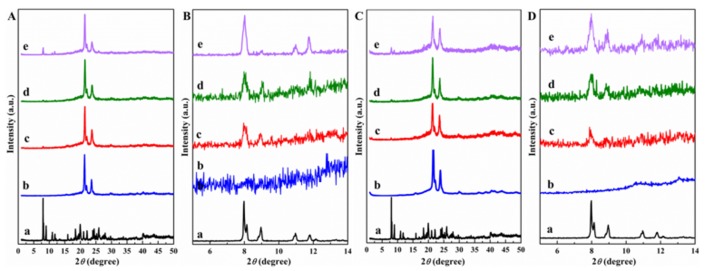
XRD patterns (**A**,**B**) solution blended, (**C**,**D**) melt mixed: (**a**) aminopropylheptaisobutyl-POSS (APIBPOSS), (**b**) Polycaprolactone (PCL), (**c**) PCL/APIBPOSS-2, (**d**) PCL/APIBPOSS-5, (**e**) PCL/APIBPOSS-10; (**B**,**D**): enlarged XRD patterns in the 2*θ* range 5–14 degrees.

The morphology and the state of the POSS dispersion in the PCL matrix were observed with the complementary techniques of XRD and TEM. Figure 2A shows XRD patterns of neat PCL and APIBPOSS, and solution blended PCL/APIBPOSS nanocomposites. A number of strong peaks were observed in the diffractogram of APIBPOSS, revealing a highly crystalline structure.

The XRD pattern of PCL showed the characteristic diffraction peaks at 2*θ* = 21.34°, 21.96°, and 23.56°, corresponding to the (110), (111), and (200) planes, respectively [27]. In the XRD diffrattograms of PCL/APIBPOSS nanocomposites, those peaks remained invariant in comparison with the PCL homopolymer, indicating that the crystalline structure of PCL was unaffected by the presence of POSS. In the enlarged XRD patterns in the 2*θ* range of 5–14° of PCL/APIBPOSS containing 2 and 5% of nanofiller (Figure 2Bc,d) a weak peak is observed at 2*θ* = 8° corresponding to the APIBPOSS crystals (Figure 2Ba). This peak centered at 2*θ* = 8° increased in intensity as the POSS content increased (Figure 2Bd–e), and more crystalline peaks corresponding to POSS were also observed. However, the peak at 2*θ* = 8.96° almost disappeared at 10 wt.% POSS content. Moreover, from the diffractograms it is observed that crystalline peaks related to POSS crystals are not as sharp as the ones for the neat APIBPOSS, indicating that the dispersed POSS crystals are not as perfect as neat POSS crystals. These observations indicate that PCL/APIBPOSS blends exhibit the characteristic features of the structures of the two separate components. These results suggest that APIBPOSS crystallizes and agglomerates in PCL matrix when it is incorporated in the polymer by solution-casting blending. The interactions between the POSS nanoparticles explain the formation of aggregates.

Figure 2C shows XRD patterns of neat extruded PCL, pure APIBPOSS and melt mixed PCL/APIBPOSS nanocomposites. As with the solution blended nanocomposites, the crystalline peak in the low angle range (2*θ* = 8°) was also detected (Figure 2D), and became more intense as the APIBPOSS concentration in the blend increased. When comparing these results with those obtained for the solution blended nanocomposites, it is clear that the melt mixed nanocomposite containing 10 wt.% of APIBPOSS exhibited a much less intense APIBPOSS crystalline peak than the solution blended counterpart, indicating that fewer crystalline aggregates of POSS molecules exist in the melt mixed sample. These results allow for the conclusion that in the solution blended nanocomposites the dispersed APIBPOSS particles aggregate together to form a crystalline structure much easier than in the melt blended samples.

**Figure 3:**
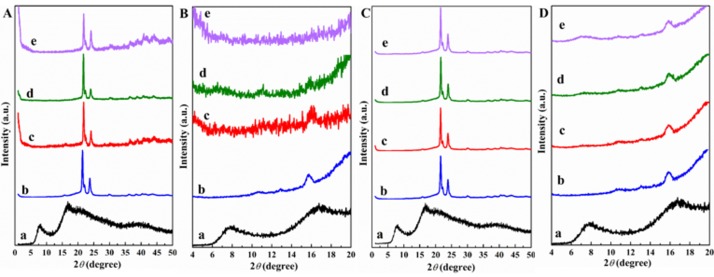
XRD patterns (**A**,**B**) solution blended, (**C**,**D**) melt mixed: (**a**) APIOPOSS, (**b**) PCL, (**c**) PCL/APIOPOSS-2, (**d**) PCL/APIOPOSS-5, (**e**) PCL/APIOPOSS-10; (**B**,**D**): enlarged XRD patterns in the 2*θ* range 5–20 degrees.

Unlike APIBPOSS, APIOPOSS is not crystalline, three amorphous halos were observed in the X-ray diffraction pattern (Figure 3Aa). Figure 3Ac–e presents XRD patterns of solution blended PCL/APIOPOSS nanocomposites. In the enlarged XRD patterns it can be observed that the signals for APIOPOSS are absent in all PCL nanocomposites containing APIOPOSS (Figure 3Bc–e). Similar results were obtained for the melt compounded PCL/APIOPOSS nanocomposites (Figure 3C,D). 

**Figure 4:**
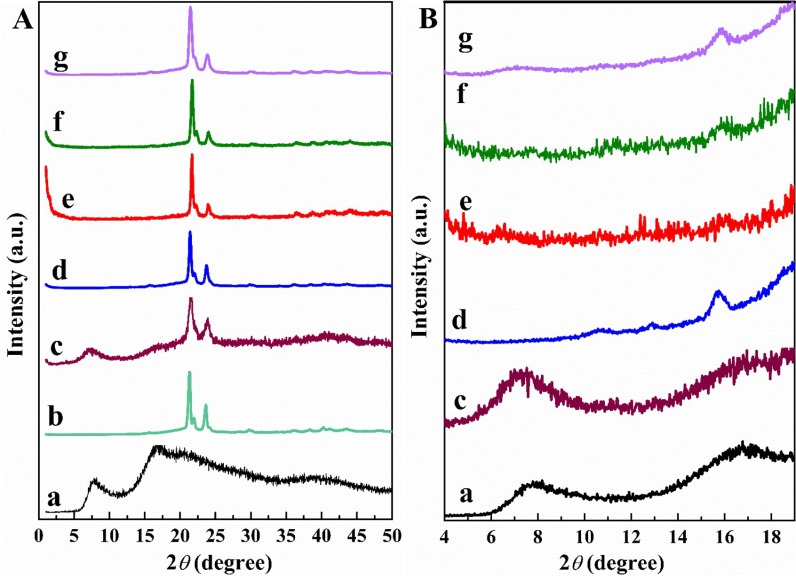
XRD patterns (**A**) solution blended: (**a**) APIOPOSS, (**b**) PCL-diol, (**c**) PIOPOSS-PCL-PIOPOSS, (**d**) PCL, (**e**) PCL/PIOPOSS-PCL-PIOPOSS-2, (**f**) PCL/PIOPOSS-PCL-PIOPOSS-5, (**g**) PCL/PIOPOSS-PCL-PIOPOSS-10; (**B**): enlarged XRD patterns in the 2*θ* range 5–20 degrees.

Figure 4 shows the XRD patterns of neat APIOPOSS and PCL diol (the precursors of the nanohybrid), telechelic hybrid PCL containing POSS, and PCL. The diffraction pattern of telechelic hybrid displays the characteristic amorphous halo of APIOPOSS at 7.7° and the characteristic diffraction peaks of PCL-diol (Figure 4c). As for solution blended PCL/PIOPOSS-PCL-PIOPOSS masterbatch nanocomposites, XRD patterns (Figure 4Ae–g) were similar to that of neat PCL (Figure 4Ad). In the enlarged XRD patterns (Figure 4Be–g) the amorphous halos of the telechelic hybrid are absent.

**Figure 5:**
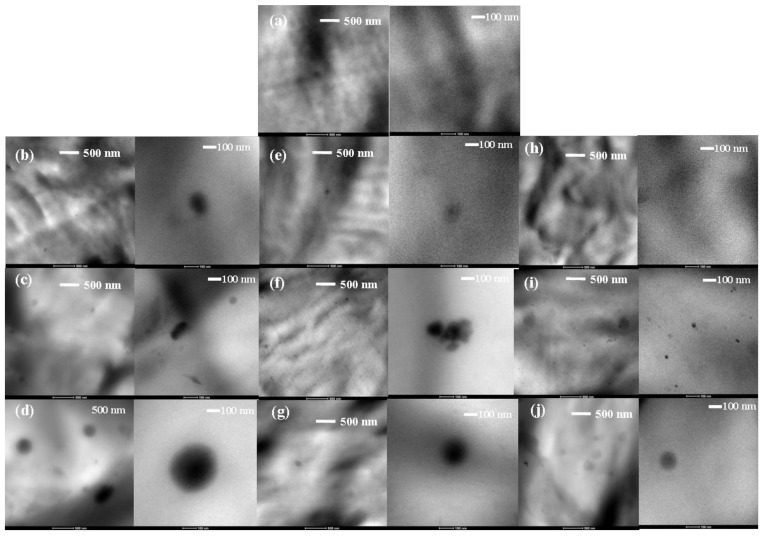
TEM micrographs of solution blended PCL/POSS nanocomposites: (**a**) PCL; (**b**) PCL/APIBPOSS-2; (**c**) PCL/APIBPOSS-5; (**d**) PCL/APIBPOSS-10; (**e**) PCL/APIOPOSS-2; (**f**) PCL/APIOPOSS-5; (**g**) PCL/APIOPOSS-10; (**h**) PCL/PIOPOSS-PCL-PIOPOSS-2; (**i**) PCL/PIOPOSS-PCL-PIOPOSS-5; (**j**) PCL/PIOPOSS-PCL-PIOPOSS-10.

**Figure 6:**
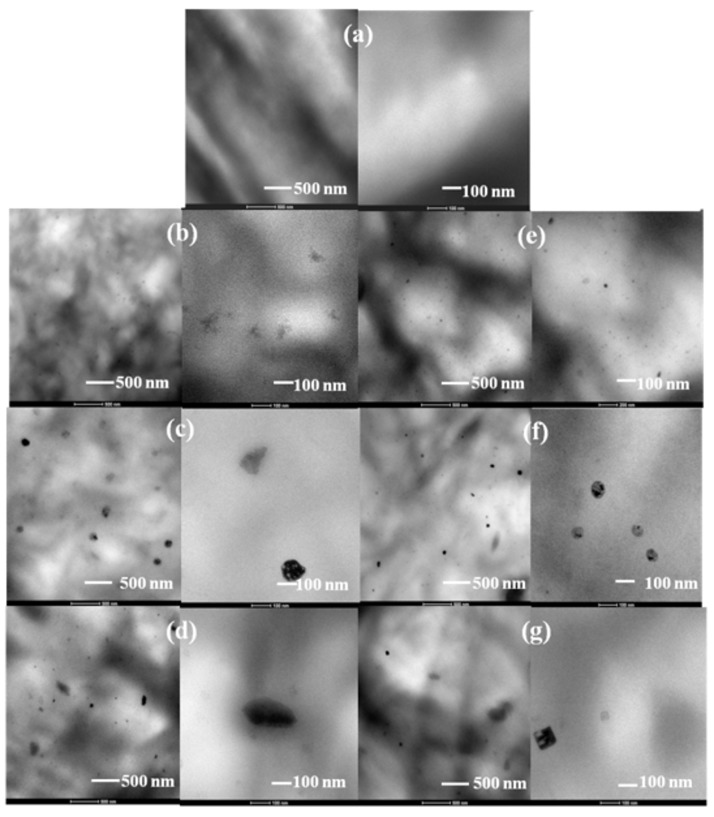
TEM micrographs of melt mixed PCL/POSS nanocomposites: (**a**) PCL; (**b**) PCL/APIBPOSS-2; (**c**) PCL/APIBPOSS-5; (**d**) PCL/APIBPOSS-10; (**e**) PCL/APIOPOSS-2; (**f**) PCL/APIOPOSS-5; (**g**) PCL/APIOPOSS-10.

The nanocomposite samples were examined with TEM to elucidate the microstructure, the dispersion state of POSS into the matrix, and the interaction level between them. Figure 5 and Figure 6 display TEM images of PCL/POSS blends and neat PCL as reference. TEM images revealed the presence of POSS aggregates. Spherical shaped particles were observed in the micrographs of solution blended PCL/POSS nanocomposites (Figure 5). The size of the POSS aggregates in nanocomposites containing APIBPOSS (Figure 5b–d) was between 130 and 380 nm, while smaller aggregates (110–270 nm) were observed for APIOPOSS nanocomposites (Figure 5e–g) and for the samples prepared by the masterbatch approach (45–135 nm at 5 and 10 wt.% nanohybrid concentrations) (Figure 5h–j). The size of the aggregates depended on the POSS type and concentration. The greatest extent of aggregation and the largest aggregates were observed at the highest POSS concentration. Amino functional-POSS molecules are dispersed in the form of aggregates within the PCL matrix. Comparing the micrographs of solution blended PCL/POSS nanocomposites, it was observed that the largest aggregates were formed in the presence of APIBPOSS, and the best dispersion was achieved when using the masterbatch.

Solubility parameters can be used for predicting the solubility of one material into another, in this case the POSS nanoparticles into the polymer matrix [18,19,28,29,30]. Furthermore, the homogeneous dispersion of the nanoparticles is largely dependent on its solubility into the polymer. Greater compatibility and better dispersion characteristics can be expected when mixing materials with similar solubility parameters when compared to those with very different solubility parameters. The literature value of the solubility parameter of the PCL, APIBPOSS, and APIOPOSS are 19.7 (J/cm^3^)^1/2^ [31], 17.5 (J/cm^3^)^1/2^ [30], and 18.6 (J/cm^3^)^1/2^ [30], respectively. APIOPOSS showed the lowest solubility parameter difference with PCL, 1.1(J/cm^3^)^1/2^, versus 2.2 (J/cm^3^)^1/2^ for APIBPOSS. Therefore, on the basis of this difference a higher solubility of PIOPOSS in the PCL matrix, that is greater compatibility, and less tendency to aggregate in comparison with APIBPOSS is expected. Considering the Flory–Huggins theory for polymer solutions and blends, the Flory–Huggins parameter *χ*_12_, included in the definition of the mixing enthalpy, can be related to the solubility parameters of two substances [32] by the relation;

(1) $$\chi_{12}=\frac{V_{M}}{RT}{\left(\delta_{1}-\delta_{2}\right)}^{2}$$

where *δ*_1_ and *δ*_2_ in our case are the solubility parameters of the PCL 1 and POSS 2, respectively, *R* and *T* are the gas constant and temperature, respectively, and *V*_M_ is a reference volume which is the molar volume of the smallest repeat unit. It is expected that the interaction between PCL and APIOPOSS would be more thermodynamically favorable than the PCL/APIBPOSS, since the values for solubility parameter are closer than that of APIBPOSS. Solubility parameter value is not determined in the case of the ditelechelic hybrid, however, an enhancement in the dispersion state would be expected since the PCL chains covalently bonded to the POSS surface could improve the compatibility between PCL and the POSS nanoparticles. This enhancement of compatibility could be due to the solubility of low molecular weight PCL chains of the hybrid into the PCL matrix. The TEM observations are in agreement with the solubility parameters.

TEM images of the melt mixed PCL nanocomposites revealed a more uniform dispersion of POSS, although POSS aggregate particles were also observed, in particular at 5 and 10 wt.% amino-POSS loading (Figure 6). The size of the particles was about 60 nm in the composites containing 2 wt.% of amino-POSS, while at higher POSS loading the size was between 110–260 nm for APIBPOSS, and between 70–125 nm for APIOPOSS based nanocomposites. Comparing the micrographs of the melt mixed and solution blended PCL/amino-POSS derivatives composites, it can be observed that the size of the POSS particle aggregates is larger for the solution blended PCL/amino-POSS nanocomposites. In the solution mixing approach, POSS nanoparticles are dispersed in the PCL solution, and nanocomposites are achieved by the evaporation of solvent. Nanoparticles tend to agglomerate due to high surface energy, even though ultrasonic mixing was used to disperse them. In conclusion, the ultrasonication step was not sufficient to properly disperse the POSS nanoparticles within the PCL matrix. In the melt compounding approach, POSS nanoparticles are incorporated into the molten polymer. The better dispersion of amino-POSS nanoparticles achieved by melt mixing as compared with the solution casting process can be due to the high shear force generated during the melt blending. The shear stress can overcome the interactions between the POSS nanoparticles and lead to the breakup of the POSS agglomerates. It has been reported that the shear stresses exerted on the polymer melt during processing helps the dispersion of fillers [33,34,35,36].

### 3.2. Effect of Melt Mixing Processing on PCL Molecular Weight

In order to assess whether degradation of PCL took place at high temperature during melt extrusion by aminolysis reaction between the primary amine function present on POSS nanocages (APIBPOSS and APIOPOSS) and the polymer matrix, molecular weight of PCL was measured. Table S1 shows the *M*_w_, *M*_n_, and the polydispersity index (*M*_w_/*M*_n_) of the neat PCL and nanocomposites extruded at 90 °C and 50 rpm for 10 min. The *M*_w_ and *M*_n_ values show that PCL did not degrade under the melt mixing conditions of nanocomposites.

### 3.3. Effect of POSS Type and Processing Method on Thermal Transitions

**Figure 7:**
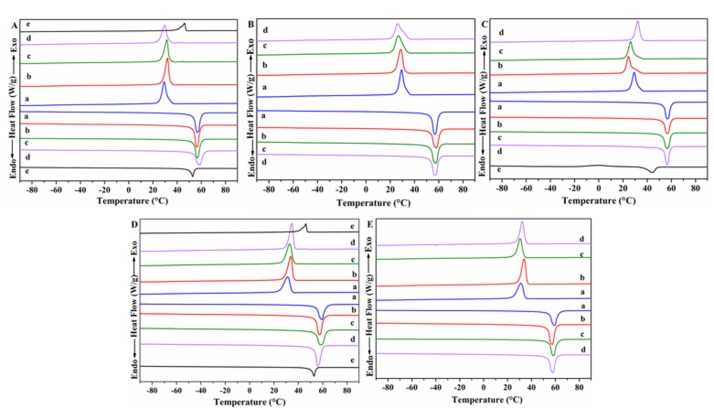
DSC thermograms of: (**A**) solution blended PCL/APIBPOSS; (**B**) solution blended PCL/APIOPOSS; (**C**) solution blended PCL/PIOPOSS-PCL-PIOPOSS; (**D**) melt mixed PCL/APIBPOSS; (**E**) melt mixed PCL/APIOPOSS: (**a**) neat PCL; (**b**) PCL/POSS-2; (**c**) PCL/POSS-5; (**d**) PCL/POSS-10; (**e**) APIBPOSS or PIOPOSS-PCL-PIOPOSS.

The determination of thermal transitions of the composites and the study of the effect of POSS type and preparation method on these transitions were conducted by DSC measurements. Figure 7 displays the DSC thermograms (second heating and first cooling scans) of neat PCL, APIBPOSS, and PCL/POSS nanocomposites. From these thermograms, the endothermic and exothermic peaks, corresponding to the melting and crystallization peaks of PCL, respectively, can be clearly seen. All the data as determined from the DSC traces are given in Table 1. The glass transition temperature (*T*_g_) of solution blended and melt processed PCL/POSS nanocomposites is roughly independent on the type and content of POSS.

Solution blended PCL nanocomposites containing 2 and 5 wt.% APIBPOSS showed similar melting temperatures (*T*_m_) as pristine PCL, while the value of the nanocomposite with 10 wt.% APIBPOSS was slightly higher (2 °C) than that of PCL. No significant differences in *T*_m_ value of PCL/APIOPOSS and PCL/POSS nanohybrid masterbatch nanocomposites were observed as compared with neat PCL. PCL/POSS nanocomposites prepared by melt mixing exhibited a slightly lower melting temperature as compared with neat extruded PCL.

As can be seen in the enlarged DSC cooling scans shown in Figure S1A and from Table 1, the crystallization temperature (*T*_c_) for solution blended nanocomposites containing 2 and 5 wt.% of APIBPOSS were slightly higher than that of neat PCL. Moreover, *T*_c_ of PCL first increased and then decreased with increasing the POSS loading in the PCL/APIBPOSS nanocomposites. However, the onset crystallization temperature (data not shown) was almost unaffected by the incorporation of APIBPOSS irrespective of POSS loading. These results indicate that although in the presence of APIBPOSS the crystallization of PCL does not start earlier as compared with neat PCL, it runs much faster, and the degree of enhancement in *T*_c_ depends of POSS concentration. This indicates that APIBPOSS nanoparticles act as nucleating agent for the crystallization of PCL. On the other hand, an additional crystallization peak at 44 °C, attributed to APIBPOSS, was also observed. This result is in accordance with that obtained by XRD analysis. *T*_c_ value of PCL was unaffected by the addition of 2 wt.% of APIOPOSS, while decreased at higher POSS loading level (Figure 7B and Figure S1B). PCL/APIOPOSS nanocomposites containing 5 and 10 wt.% exhibited a wider crystallization process as compared with that of neat PCL. These results suggest that APIOPOSS did not act as nucleating agent. *T*_c_ value of nanocomposites containing 2 and 5 wt.% PIOPOSS-PCL-PIOPOSS hybrid was lower than that of neat PCL, while the value for the blend containing 10 wt.% was higher than that of PCL (Figure 7C and Figure S1C). The onset crystallization temperature (data not shown) was almost unaffected by the incorporation of this nanohybrid irrespective of POSS loading. These results indicate that the crystallization of PCL runs much faster in the presence of 10 wt.% of ditelechelic hybrid, even though the crystallization of PCL does not start earlier as compared with neat PCL. 

The *T*_c_ values of PCL/APIBPOSS nanocomposites prepared by melt mixing are slightly higher than that of PCL (Figure 7D and Figure S1E), and the crystallization peak at 45 °C attributed to POSS was observed in the blend containing 10 wt.% APIBPOSS. The onset crystallization temperature (data not shown) of melt processed PCL/APIBPOSS composites were shifted toward higher temperatures indicating that those nanoparticles act as nucleating agents. The exothermic peak of the PCL crystallization process was narrower in PCL/APIBPOSS nanocomposites. The blends containing 2 and 10 wt.% APIOPOSS also exhibited a slightly higher *T*_c_ value than that of neat PCL (Figure 7E and Figure S1F), a narrower exothermic peak of crystallization, and higher onset crystallization temperatures. These results indicate that APIOPOSS nanoparticles acted as nucleating agents for the crystallization process of PCL.

As a conclusion, the crystallization of PCL is affected by the presence of POSS, the type of POSS derivative, the POSS content, and the preparation method. For the blends prepared by solution-casting, APIBPOSS acts as a nucleating agent for the crystallization of PCL, while the nucleating effect is inexistent for APIOPOSS, and a high content of the PIOPOSS-PCL-PIOPOSS hybrid is necessary to act as nucleating agent. If nanocomposites are prepared by melt compounding, APIBPOSS acts as nucleating agent, and this effect it is observed only at 2 and 10 wt.% APIOPOSS loading.

The crystallization enthalpy of nanocomposites decreased gradually by increasing the POSS content. For the solution blended nanocomposites, Δ*H_c_* of PCL increased slightly upon addition of 2 wt.% APIBPOSS and then decreased with a further increase in POSS content. The same trend was observed for nanocomposites with the ditelechelic hybrid masterbatch. However, the addition of 2 and 5 wt.% of the APIOPOSS had no effect on Δ*H_c_* value of PCL, and the incorporation of 10 wt.% of POSS led to a reduction in that value. The POSS nanoparticles reduce the crystalline order during crystallization and reduces Δ*H_c_* of PCL. On the other hand, in the melt mixed nanocomposites, the addition of the amino-POSS derivatives led to a slight increase in Δ*H_c_* of PCL.

The percentage crystallinity (% *X*_c_) of PCL was calculated according to the following relation,

(2) $$\chi_{c}=\left[\frac{\Delta H_{m}}{\Delta H_{m}^{0}\times \left(1-\frac{\%wt_{filler}}{100}\right)}\right]\times 100$$

where, Δ*H_m_* is the enthalpy of fusion, Δ*H^0^_m_* is the enthalpy of fusion of a perfect PCL crystal (142 J g^−1^) [37], and wt.% filler is the total weight percentage of POSS derivative. For the solution casting samples, the addition of 2 wt.% APIBPOSS led to an increase in the degree of crystallinity of PCL, while it decreased with a further increase in POSS content. This can be due to the formation of aggregates and hence difficulty in transportation of PCL chains to crystal growing surface. The crystallinity of the solution blended PCL/APIOPOSS nanocomposites was almost the same than that of neat PCL. *X_c_* value increased as ditelechelic hybrid content increased, the composite containing 10 wt.% of POSS exhibited a slightly higher degree of crystallinity than PCL. The incorporation of APIBPOSS by melt compounding led to an increase in the degree of crystallinity of PCL that increased with the POSS content. For the APIOPOSS based nanocomposites the addition of 2 and 10 wt.% of POSS led to an increase in the crystallinity of PCL, while the incorporation of 5 wt.% of POSS had no effect.

### 3.4. Effect of POSS Type and Processing Method on Thermal Stability

Thermogravimetric analysis (TGA) was carried out in an inert (N_2_) and oxidative (air) atmosphere to study the thermal and thermo-oxidative stability of PCL/POSS nanocomposites. Table 2 shows the thermal decomposition temperatures for 5% weight loss (*T*_5%_), the temperature of the maximum loss rate (*T_max_*) and the fraction of solid residue at 750° C of the thermograms.

**Figure 8:**
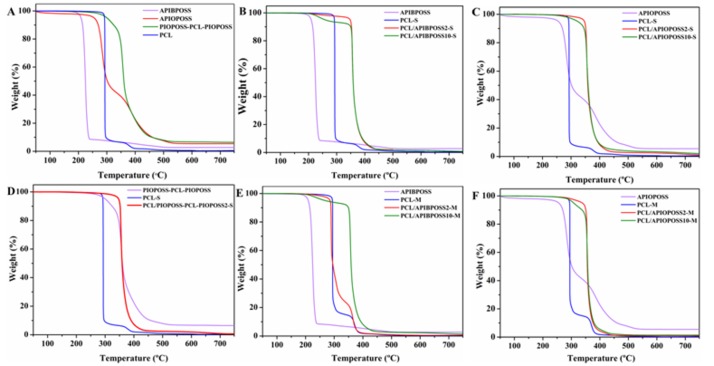
TGA curves of: (**A**) nanofillers and neat PCL; (**B**–**D**), solution blended nanocomposites; (**E**,**F**) melt mixed nanocomposites in N_2_ atmosphere.

Figure 8 displays TGA curves in N_2_ atmosphere of neat POSS derivatives, neat PCL and PCL/POSS derivatives nanocomposites, whilst the derivative thermogravimetric (DTG) curves are shown in Figure S2. 

APIBPOSS decomposed in a one step process in the 170–260 °C temperature range, the weight loss of APIOPOSS took place in a two-step process in the 200–550 °C temperature range, and PIOPOSS–PCL-PIOPOSS exhibited a single stage degradation process in the temperature range 200–480 °C. As reported in Table 2, *T*_5%_ value of PCL was higher than that of neat amino-POSS molecules and slightly lower than that of PIOPOSS-PCL-PIOPOSS hybrid. The solid residue amount at 750 °C was approximately 2.8% for APIBPOSS, 5.5% for APIOPOSS, and 6.4% for PIOPOSS-PCL-PIOPOSS. Thermal stability of neat POSS in nitrogen was observed to decrease in order: PIOPOSS-PCL-PIOPOSS > APIOPOSS > APIBPOSS (Figure 8A). The longer the alkyl chain of substituents in the POSS molecule, the higher the *T*_5%_ value (Table 2). Similar results were obtained in the literature [23,38]. On the other hand, PCL decomposed completely in two steps in the temperature range of 280–420 °C. When PCL was blended with APIBPOSS by solution-casting method, all the samples decomposed completely in two steps in the temperature range of 180–450 °C (Figure 8B). The weight loss of the first stage, in the range of 180–310 °C, was between 2 and 7% and increased with increasing the POSS content, and can be attributed to the decomposition of APIBPOSS, since this filler decomposes in this temperature range (Figure 8A and Figure S2). Improved thermal stability was observed for the nanocomposites with 2 and 5 wt.% of APIBPOSS, being the onset temperature of the degradation more than 50 °C higher than that of the pure PCL, whereas the *T*_5%_ value of the nanocomposite containing 10 wt.% was almost 30 °C lower than that of neat polymer matrix, this can be ascribed to the decomposition of APIBPOSS aggregates, larger than those of blends containing 2% and 5% POSS. A significant increase in *T_max_* value of the nanocomposites was observed in comparison with neat PCL.

All solution blended PCL/APIOPOSS nanocomposite samples decomposed completely in a single step in the temperature range of 300–450 °C (Figure 8C). The incorporation of APIOPOSS enhanced the thermal stability of PCL, being the values of *T*_5%_ improved by more than 40 °C. The *T*_5%_ of the nanocomposites decreased continuously with POSS content. A significant increase in *T_max_* values of the nanocomposites in comparison with neat PCL was observed. The addition of PIOPOSS-PCL-PIOPOSS led to an enhancement of thermal stability of PCL (Figure 8D). A noticeable increase was observed in the *T*_5%_ value (55 °C). *T_max_* value was independent of the POSS content. 

The improvement in the thermal stability of the PCL/POSS nanocomposites can be explained by the formation of interactions between POSS and PCL matrix. The decline in thermal stability of PCL/amino-POSS nanocomposites as POSS concentration increases can be ascribed to the poorer dispersion level of nanoparticles in the polymer matrix, since larger aggregates were formed. The reduction of the thermal stability in the PCL/APIBPOSS-10-S can be associated to the poorer dispersion of the nanoparticles at this concentration level, as well as to the lower thermal stability of neat APIBPOSS as compared to PCL. The dependence of the thermal stability of POSS containing polymer nanocomposites on the type and content of POSS as well as on dispersion level of POSS nanoparticles in the polymer matrix has been reported in the literature [39].

Neat extruded PCL and melt blended PCL/APIBPOSS sample containing 2 wt.% APIBPOSS displayed similar degradation profiles, two decomposition steps in the temperature range of 220–400 °C (Figure 8E). The nanocomposites with 5 and 10 wt.% APIBPOSS decomposed in two steps, in the temperature range 200–325 °C and 325–460 °C, respectively. The weight loss in the first stage was 4% and 8% for the blend with 5 wt.% POSS and 10 wt.% POSS, respectively. This step can be attributed to the decomposition of the amino-POSS filler. For the melt mixed nanocomposites containing 5 wt.% of APIBPOSS, the onset temperature of the degradation is about than 50 °C higher than that of the pure PCL, whereas the *T*_5%_ value of the nanocomposites containing 2 and 10 wt.% APIBPOSS are slightly lower than that of neat polymer matrix. The TGA weight loss curves of the melt mixed PCL/APIOPOSS nanocomposites with 2 and 5 wt.% POSS exhibited a single decomposition step between 315 °C and 420 °C, while in the thermogram of the blend with 10% POSS two steps were observed in the 275–330 °C and 330–400 °C temperature range, respectively. The weight loss in the first step was about 8%, and can be attributed to APIOPOSS decomposition. The *T*_5%_ value of the PCL/APIOPOSS nanocomposites was higher than that of neat PCL (Table 2 and Figure 8F), and decreased with increasing APIOPOSS content. A significant increase in *T_max_* value of the APIBPOSS and APIOPOSS based nanocomposites was observed as compared with neat PCL.

**Figure 9:**
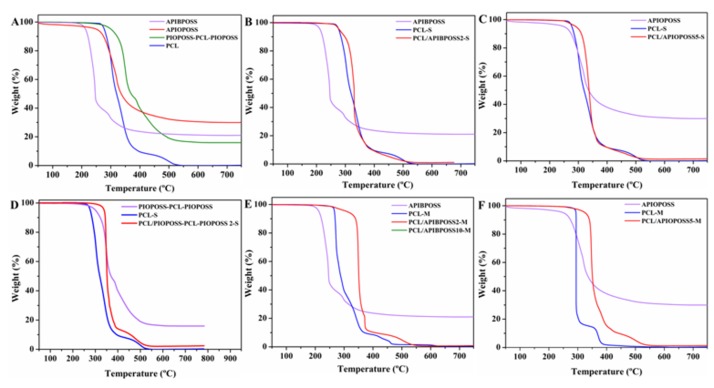
TGA curves of: (**A**) nanofillers and neat PCL; (**B**–**D**) solution blended nanocomposites; (**E**,**F**) melt mixed nanocomposites in O_2_ atmosphere.

The TG curves in air atmosphere for POSS derivatives, neat PCL and PCL/POSS derivative nanocomposites are displayed in Figure 9, whilst DTG curves are shown in Figure S3. When heated in oxidative atmosphere, APIOPOSS showed a single step process in the 225–550 °C temperature range (Figure 9A), whereas APIBPOSS and PIOPOSS–PCL-PIOPOSS exhibited a two-step process in the 160–400 °C and 200–600 °C temperature range, respectively. Thermal stability in air of neat POSS derivatives decreased in the order: PIOPOSS-PCL-PIOPOSS > APIOPOSS > APIBPOSS. As in case of thermal stability in nitrogen atmosphere, the longer the alkyl chain of substituents in the POSS molecule, the higher the thermo-oxidative stability. The solid residue obtained from the degradations of POSS derivatives appears because of the formation of silica, SiO_2_, and higher residue appeared in oxygen than in nitrogen as expected [38,40]. When POSS molecules are added to a polymer matrix they self-segregate to the polymer surface due to the low surface energy of Si atom, upon heating they form a ceramic layer which prevents the heat transfer to the sample and the permeability of volatile products from generating in the degradation process [38,41]. The residue left by APIBPOSS and APIOPOSS after thermo-oxidative decomposition amounted to 21.1% and 30%, respectively. Those values are lower than the theoretical expected value of 54.8% and 38%, respectively, with the total conversion of the inorganic cage to SiO_2_. The lower residue for both amino-POSS derivatives is due to the sublimation of a part of the cubic cage of amino-POSS. The amount of residue left by APIOPOSS is 8% lower than expected, versus 33.7% for APIBPOSS, indicating that APIOPOSS is less susceptible to sublimation than APIBPOSS.

The presence of oxygen led to a faster degradation of the PCL and solution blended PCL/POSS nanocomposites, than in inert conditions (Table 2), the oxidizing atmosphere accelerates the degradation process. The *T*_5%_ value of neat PCL was lowered by 15 °C owing to the simultaneous effect of heat and reaction with oxygen. *T*_5%_ values of nanocomposites containing 2 wt.% amino-POSS were about 70 °C lower than those under nitrogen atmosphere. When PIOPOSS-PCL-PIOPOSS hybrid was blended with PCL, *T*_5%_ value was 10 °C lower than that under nitrogen atmosphere. With regard to the thermo-oxidative behavior of the solution blended PCL/APIBPOSS nanocomposites, the onset decomposition temperatures were similar to that of neat PCL (Figure 9B), while *T*_5%_ values of the PCL/ditelechelic hybrid masterbatch nanocomposites (Figure 9D), and that of those containing 5 and 10 wt.% APIOPOSS were higher than that of PCL (Figure 9C). On the other hand, *T*_5%_ values of all melt mixed nanocomposites increased by about 40–55 °C, in comparison with neat PCL (Table 2, Figure 9E,F). A noticeable improvement in *T*_5%_ values was observed for melt blended amino-POSS based nanocomposites as compared with those prepared by solution mixing, which can be attribute to the more uniform distribution and smaller aggregates of the filler.

On the basis of the above results, it can be concluded that thermo-oxidative stability of PCL improved with the incorporation of the masterbatch (PIOPOSS-PCL-PIOPOSS) and APIOPOSS (at content greater than 2 wt.%) by solution mixing, while it remained unchanged with the incorporation of APIBPOSS. However, the thermo-oxidative stability of PCL was enhanced by the incorporation amino-POSS derivatives by melt mixing. The ditelechelic hybrid masterbatch provided the greatest improvement both in thermal and thermo-oxidative stability of PCL for the entire filler content range studied, that can be explained by the better compatibility with PCL chains, which results in a better extent of dispersion (smaller size aggregates). The enhancement in thermal stability of nanocomposite films can be ascribed to the thermal insulator and mass transport barrier effect of POSS nanoparticles.

### 3.5. Effect of POSS Type and Processing Method on Mechanical Properties

**Figure 10:**
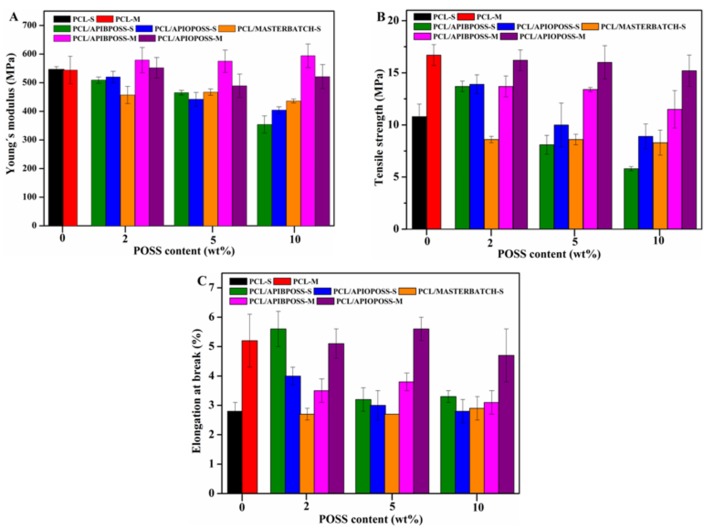
Mechanical properties of PCL/POSS nanocomposite films: (**A**) Young´s modulus; (**B**) Tensile strength; and (**C**) Elongation at break.

The Young´s modulus, tensile strength and strain at break were determined from experimental tensile stress-strain curves shown in Figure S4. The results as a function of the POSS content and the processing method are shown in Figure 10.

The results obtained from the solution blended nanocomposites showed a decrease in elastic modulus of the nanocomposite samples as compared to the pure PCL (Figure 10A), and its value was affected by the POSS type and content, decreasing as amino-POSS derivative content increased and remained almost unchanged with PIOPOSS-PCL-PIOPOSS concentration. Comparing the POSS type, the blend containing 10 wt.% APIBPOSS showed the lowest Young´s modulus, while that containing 2 wt.% APIOPOSS exhibited the highest one. The decrease in elastic modulus was between 7 and 35% for the PCL/APIBPOSS system, between 5 and 25% for the PCL/APIOPOSS system, and between 15 and 20% for the PCL/PIOPOSS-POSS-PIOPOSS nanohybrid nanocomposites. The Young´s modulus in the blend containing 2 wt.% of the PIOPOSS-POSS-PIOPOSS masterbatch is lower than that value for the blends containing 2 wt.% of amino-POSS derivatives. The results indicate that the stiffness of PCL matrix is reduced by the incorporation of APIBPOSS, APIOPOSS. and the PIOPOSS-PCL-PIOPOSS masterbatch via solution blending. This decrease in stiffness can be associated to the decrease in the degree of crystallinity of the PCL matrix with respect to neat PCL only for PCL/APIBPOSS-10-S nanocomposite. Baldi et al. [42] reported the effect of the length of POSS alkyl chain on the mechanical behavior of POSS/PP blends. In their study, they used octamethyl-POSS, octaisobutyl-POSS, and octaisooctyl-POSS. The authors suggested that POSS behave as particles having a siliceous hard-core surrounded by a hydrocarbon soft-shell, which limits the stress transfer from the polymer matrix to the core in dependence on the length of the alkyl groups. The thickness of the shell that is determined by the length of the R side groups of POSS molecules was pointed out as the key factor in permitting the rigid part of the particle to express a reinforcing effect. POSS bearing long substituents behave as rubbery inclusions. Moreover, since the inorganic cage promote the reinforcing action, they stated that the amount of inorganic material dispersed in the polymer matrix must be taken into account, that is, with the same POSS content the inorganic amount depends on the contribution of the organic fraction. The authors found an enhancement in the Young´s modulus of PP containing 10 wt.% of octamethyl-POSS, and lack of stiffness enhancement in the case of PP blends containing either octaisobutyl- or isooctyl-POSS. 

The difference between APIBPOSS and APIOPOSS is the length of the alkyl chains bounded to the Si atom, four and eight carbon atoms for APIBPOSS and APIOPOSS, respectively. An identical siliceous hard core is enveloped by alkyl chains that constitute a softer outer shell of variable thickness. APIBPOSS nanoparticles have the thinnest soft shell, whereas APIOPOSS has the thickest one. In the PIOPOSS-PCL-PIOPOSS nanohybrid in addition to eight alkyl chains there is a PCL chain grafted. The amount of inorganic material dispersed in the PCL/PIOPOSS-PCL-PIOPOSS blends is lower than that dispersed in the PCL/amino-POSS systems with the same POSS content. Our results confirm the observation of Baldi et al. [42].

The solution blended nanocomposites containing 2 wt.% of APIBPOSS and APIOPOSS displayed an increase in tensile strength at break of about 28%, with respect to PCL (Figure 10B), whilst a higher content of amino-POSS derivatives resulted in a reduction (about 25–45% for APIBPOSS based nanocomposites, and 8–18% for the PCL/APIOPOSS composites). The tensile strength of PCL/PIOPOSS-PCL-PIOPOSS composites were independent of the hybrid content and broke at lower stress than PCL, about 20% reduction was observed. The decline of tensile strength with increasing amino-POSS content could be ascribed to the formation of larger aggregates. At low amino-POSS content, partial tensile strain can be transferred to the hard core of POSS nanoparticles dispersed in the PCL matrix under tensile stress, which leads to the increase of tensile strength. Further addition of filler results in more and larger agglomerates of POSS in PCL and they behave as defects that lead to a decrease of tensile strength. The lower tensile strength attained in the APIBPOSS based nanocomposites as compared with those based on APIOPOSS can be attributed to the larger size of the agglomerates formed in the first case, which act as crack propagation site and lead to the failure under stress. The lack of enhancement in tensile strength in the PCL blends containing PIOPOSS-PCL-PIOPOSS hybrid can be explained by the lower amount of siliceous hard core dispersed in the polymer matrix as compared with the amino-POSS derivatives.

The addition of APIBPOSS nanoparticles induced an increase in the elongation at break of PCL (Figure 10C), 100% with 2 wt.% of APIBPOSS that is an increase in the ductility of PCL. An increase in APIBPOSS concentration above 2% resulted in a decrease in that value. The elongation at break of PCL in the presence of 2 wt.% of APIOPOSS increased by about 40%, about 7% when the APIOPOSS content was 5 wt.%, and a further increase in the nanofiller content had no effect. On the contrary, the addition of PIOPOSS-PCL-PIOPOSS to PCL had almost no effect on the elongation at break of neat polymer matrix. The presence of amino-POSS particles in PCL decreases the brittleness of the material.

The Young´s modulus of PCL was almost unaffected by the addition of amino-POSS by melt mixing. On the other hand, the tensile strength of all melt mixed PCL/APIBPOSS nanocomposites was lower than that of PCL, the reduction was between 20% and 30%. The tensile strength of PCL was almost unaffected by the addition of APIOPOSS. The addition of all APIBPOSS nanoparticles induced a reduction between 30 and 40% in the elongation at break of PCL, whilst the incorporation of APIOPOSS induced only a slight change.

The type of POSS derivative and content, and the preparation method have effect on the mechanical properties of PCL/POSS based nanocomposites, due to differences in compatibility and dispersion state. However, the better dispersion state of POSS achieved in the melt mixed PCL/amino-POSS nanocomposites is not enough to achieve good reinforcement, as strong interfacial interaction between the matrix and the filler is also necessary.

### 3.6. Effect of POSS Type on Surface Properties

**Figure 11:**
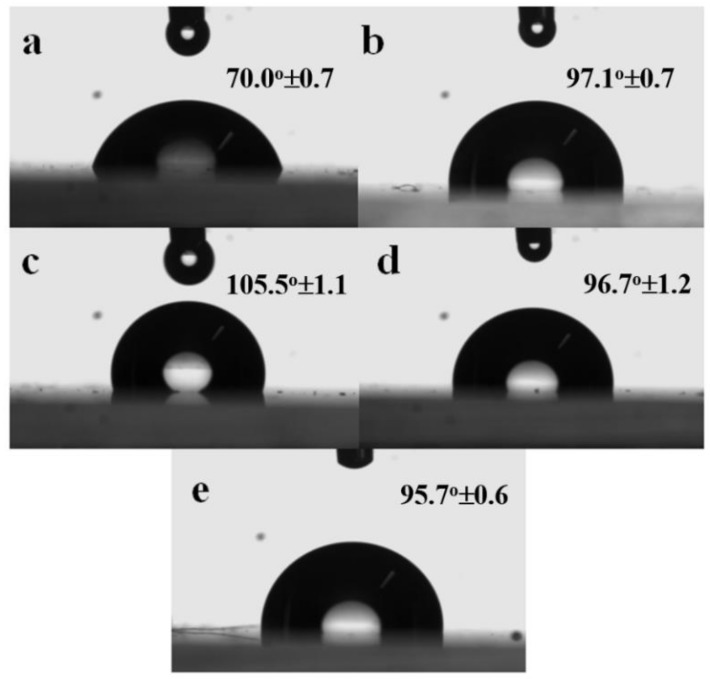
Digital images of water contact angle of (**a**) PCL; (**b**) PIOPOSS-PCL-PIOPOSS; (**c**) PCL/APIBPOSS-5; (**d**) PCL/APIOPOSS-5; and (**e**) PCL/PIOPOSS–PCL-PIOPOSS-5 nanocomposites.

Studies reported in the literature indicated that hydrophobicity of polymer matrices increased on incorporation of POSS nanoparticles [43,44]. The hydrophobicity of the nanocomposite surface as a function of POSS type and content was assessed by contact angle measurements with water. The water contact angles (WCA) were measured ten times for each specimen, and the average values are presented in Table S2. The water contact angle images for neat PCL, PIOPOSS-PCL-PIOPOSS hybrid, and PCL/POSS derivatives nanocomposites are displayed in Figure 11. 

For neat PCL the contact angle was 70.0°, and increased dramatically with loading of POSS derivatives, by 51%, 39%, and 37% for APIBPOSS, APIOPOSS, and ditelechelic hybrid, respectively (Table S2). WCA were almost independent of POSS content. The PCL/APIBPOSS based nanocomposites exhibited the highest value of contact angle. Therefore, from the results it can be concluded that the surface hydrophobicity of the PCL improves by the incorporation of POSS nanoparticles. Surface energy and roughness are some of the factors that affect contact angle [45]. POSS derivatives are organic–inorganic hybrid materials containing a central hydrophobic inorganic core silicon–oxygen–silicon surrounded by organic groups located at the corners of the octahedral siloxane cube. The increase in the contact angle values for PCL/POSS can be explained to the migration of POSS to the external surface of the nanocomposite films due to the low surface energy of the Si atom, thus making the surface more hydrophobic [46,47]. Furthermore the migration of the POSS aggregates to the surface can increase the surface roughness [36,43,47], thereby increasing the water contact angles. The highest water contact angle exhibited by PCL/APIBPOSS nanocomposites can be due to worst dispersion state of POSS and the larger aggregates formed in the presence of this POSS derivative.

## 4. Conclusions

Three polyhedral oligomeric silsesquioxane (APIBPOSS, APIOPOSS, and PIOPOSS-PCL-PIOPOSS masterbatch) were incorporated into PCL matrix at different contents via solution casting and melt mixing. Morphology, thermal, and mechanical properties depended on the alkyl chain length of the nonreactive organic substituents attached to the corner silicon atoms, the presence of PCL chains covalently attached to POSS nanoparticles, POSS concentration, and preparation method.

Spherical POSS aggregates of submicron size were observed within the PCL matrix, remaining the crystalline nature of APIBPOSS. Melt mixing led to a better dispersion state of amino-POSS nanoparticles than solution blending. The best dispersion in solution blended nanocomposites was achieved when using the masterbatch method. The difference in the dispersion level for different POSS derivative was attributed to their differences in solubility parameters.

A nucleating effect was observed during crystallization process of the melt mixed nanocomposites due to the APIBPOSS and APIOPOSS nanoparticle presence, while only APIBPOSS and high levels of ditelechelic hybrid masterbatch behaved as nucleating agents in the solution blended composites. The nanocomposites obtained by solution blending were more thermally stable than neat PCL. The thermo-oxidative stability of PCL improved with loading of APIOPOSS and ditelechelic hybrid masterbatch by solution mixing, and amino-POSS derivatives by melt blending. 

The Young´s modulus of PCL decreased with the incorporation of the three types of POSS by solution-casting method, while remained almost constant for the melt mixed nanocomposites. The incorporation of POSS nanoparticles to PCL by solution blending led to a decrease in the material stiffness and brittleness. However, in the extruded blends the incorporation of APIBPOSS nanoparticles led to more brittle materials. The addition of POSS nanoparticles to PCL render the surface hydrophobic due to presence of filler on the surface.

In this study, we have demonstrated that by controlling the POSS structure and content, and the processing method the properties of POSS/PCL nanocomposites may be tailored.

## Figures and Tables

**Table 1:** DSC data for PCL and PCL/POSS composites.

Sample	*T_g_* (°C)	*T_c_* (°C)	Δ*H_c_* (J/g)	*T_m_* (°C)	Δ*H_m_* (J/g)	*X*_c_ (%)
APIBPOSS		46.1	18.5	52.9	19.7	
PIOPOSS-PCL-PIOPOSS	−62.0	0	18.5	44.5	28.1	
PCL-S	−61.0	29.3	64.9	56.5	70.2	49.4
PCL/APIBPOSS-2-S	−61.2	32.0	67.9	56.3	72.1	51.8
PCL/APIBPOSS-5-S	−61.8	31.444.7	60.5	56.6	66.1	49.0
PCL/APIBPOSS-10-S	−59.6	29.7 44.0	57.2	58.5	57.9	45.3
PCL/APIOPOSS-2-S	−60.7	28.7	65.5	58.0	68.2	49.0
PCL/APIOPOSS-5-S	−62.0	26.7	65.3	57.5	68.5	50.8
PCL/APIOPOSS-10-S	−60.6	26.0	61.4	57.4	63.5	49.7
PCL/PIOPOSS-PCL-PIOPOSS-2-S	−61.3	24.5	67.4	57.0	69.7	50.1
PCL/PIOPOSS-PCL-PIOPOSS-5-S	−60.2	26.3	65.0	57.2	68.5	50.8
PCL/PIOPOSS-PCL-PIOPOSS-10-S	−59.7	32.3	63.4	56.4	69.3	54.2
PCL-M	−58.7	31.2	56.2	59.3	61.2	43.1
PCL/APIBPOSS-2-M	−61.0	33.7	64.5	57.3	70.8	50.9
PCL/APIBPOSS-5-M	−60.6	32.8	62.3	58.5	69.1	51.2
PCL/APIBPOSS-10-M	−60.9	34.7 45.5	61.0	55.9	68.1	53.3
PCL/APIOPOSS-2-M	−60.1	33.9	68.2	57.3	69.7	50.1
PCL/APIOPOSS-5-M	−58.4	30.5	56.0	58.2	56.5	41.9
PCL/APIOPOSS-10-M	−60.5	32.2	62.0	57.9	64.5	50.5

**Table 2:** Thermogravimetric analysis (TGA) data for PCL, PCL/APIBPOSS, PCL/APIOPOSS, PCL/PIOPOSS-PCL-PIOPOSS composites, neat APIBPOSS, APIOPOSS, and PIOPOSS–PCL-PIOPOSS.

	*T_5_* (°C)		*T_max_* (°C)		Residue (%)	
Sample	N_2_	O_2_	N_2_	O_2_	N_2_	O_2_
APIBPOSS	210	221	224	245	2.8	21.1
APIOPOSS	255	250	283 359	306 320	5.5	30.0
PIOPOSS-PCL-PIOPOSS	301	300	356	349 392	6.4	16.0
PCL-S	292	277	293	320 311	0.6	0.3
PCL/APIBPOSS-2-S	348	279	356	330	0.5	1.1
PCL/APIBPOSS-5-S	346	275	357	350	0.5	1.4
PCL/APIBPOSS-10-S	264	274	357	353	0.8	2.3
PCL/APIOPOSS-2-S	347	269	355	301	1.1	1.1
PCL/APIOPOSS-5-S	336	292	357	338	1.0	1.5
PCL/APIOPOSS-10-S	332	297	355	350	1.7	2.4
PCL/PIOPOSS-PCL-PIOPOSS-2-S	347	339	355	348	0.8	0
PCL/PIOPOSS-PCL-PIOPOSS-5-S	345	335	355	349	0.9	1.3
PCL/PIOPOSS-PCL-PIOPOSS-10-S	343	332	353	349	1.1	2.0
PCL-M	294	267	294	271 342	0.5	0.5
PCL/APIBPOSS-2-M	286	311	289	349	0.8	0.9
PCL/APIBPOSS-5-M	343	318	358	351	0.9	1.7
PCL/APIBPOSS-10-M	277	293	357	351	1.3	1.6
PCL/APIOPOSS-2-M	339	324	355	347	1.0	1.1
PCL/APIOPOSS-5-M	332	326	354	347	2.0	1.5
PCL/APIOPOSS-10-M	307	325	356	349	1.5	2.4

## Supplementary Materials

The following are available online at http://www.mdpi.com/2073-4360/11/1/33/s1; Table S1: Molecular weights of PCL and its nanocomposites; Figure S1: DSC enlarged cooling scans A, B and C: solution blended nanocomposites; D and E: melt mixed nanocomposites: (a) PCL, (b) 2 wt.% POSS, (c) 5 wt.% POSS, and (d) 10 wt.% POSS.; Figure S2: DTG curves of: (A) nanofillers and neat PCL; (B), (C), and (D) solution blended nanocomposites; (E) and (F) melt mixed nanocomposites in N2 atmosphere; Figure S3: DTG curves of: (A) nanofillers and neat PCL; (B), (C), and (D) solution blended nanocomposites; (E) and (F) melt mixed nanocomposites in O2 atmosphere; Figure S4: Stress-strain curves A, B, and C: solution blended nanocomposites; D and E: melt mixed nanocomposites: (a) PCL, (b) 2 wt.% POSS, (c) 5 wt.% POSS, and (d) 10 wt.% POSS; Table S2: Contact angles of neat PCL, PIOPOSS-PCL-PIOPOSS, and PCL/POSS nanocomposites.
