# Supplementary material for: PCL/POSS Nanocomposites: Effect of POSS Derivative and Preparation Method on Morphology and Properties
Source: Polymers (Basel). 2018 Dec 26;11(1):33. doi: 10.3390/polym11010033 (PMC6401919; doi:10.3390/polym11010033)
Supplement: Supplementary file 1 [file polymers-11-00033-s001.pdf]

polymers

MDPI

Supplementary Materials

PCL/POSS Nanocomposites: Effect of POSS Derivative and Preparation Method on Morphology and Properties

Mónica Cobos, Johnny R. Ramos, Dailyn J. Guzmán, M. Dolores Fernández and M. Jesús Fernández[1,*]

Department of Polymer Science and Technology, Faculty of Chemistry, University of the Basque Country (UPV/EHU), Pº Manuel Lardizábal 3. 20018 San Sebastián. Spain; monica.cobos@ehu.es (M.C.); johnram@hotmail.com (J.R.R.); daylyngm@yahoo.es (D.J.G.); mariadolores.fernandez@ehu.es (M.D.F.)

* Correspondence: mjesus.fernandez@ehu.es (M.J.F.); Tel.: +34-943-01-5353

Table S1. Molecular weights of PCL and its nanocomposites.

Sample	M_n (g/mol)	M_w (g/mol)	M_w/M_n
PCL	36500	54300	1.49
PCL-M	36700	52400	1.43
PCL/APIBPOSS-2-M	38200	54500	1.43
PCL/APIBPOSS-5-M	38000	54000	1.42
PCL/APIBPOSS-10-M	41000	57000	1.38
PCL/APIOPOSS-2-M	37900	54000	1.43
PCL/APIOPOSS-5-M	37800	53500	1.42
PCL/APIOPOSS-10-M	41600	56100	1.35

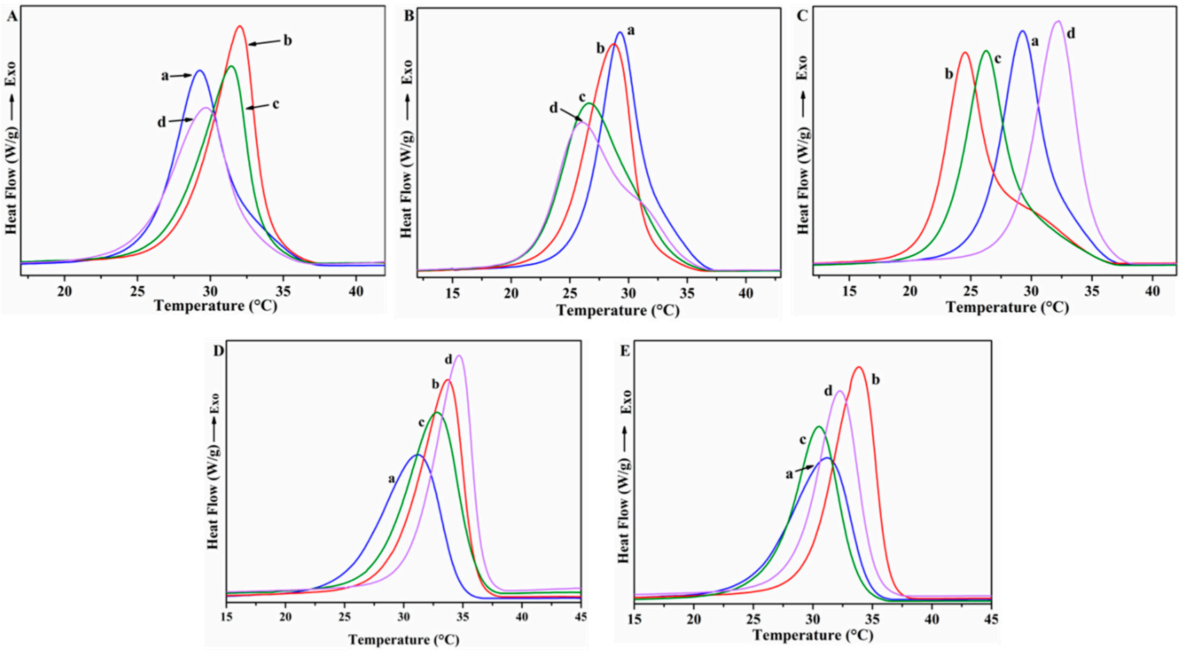

Figure S1: DSC enlarged cooling scans A, B and C: solution blended nanocomposites; D and E: melt mixed nanocomposites; (a) PCL, (b) 2 wt% POSS, (c) 5 wt% POSS, and (d) 10 wt% POSS.

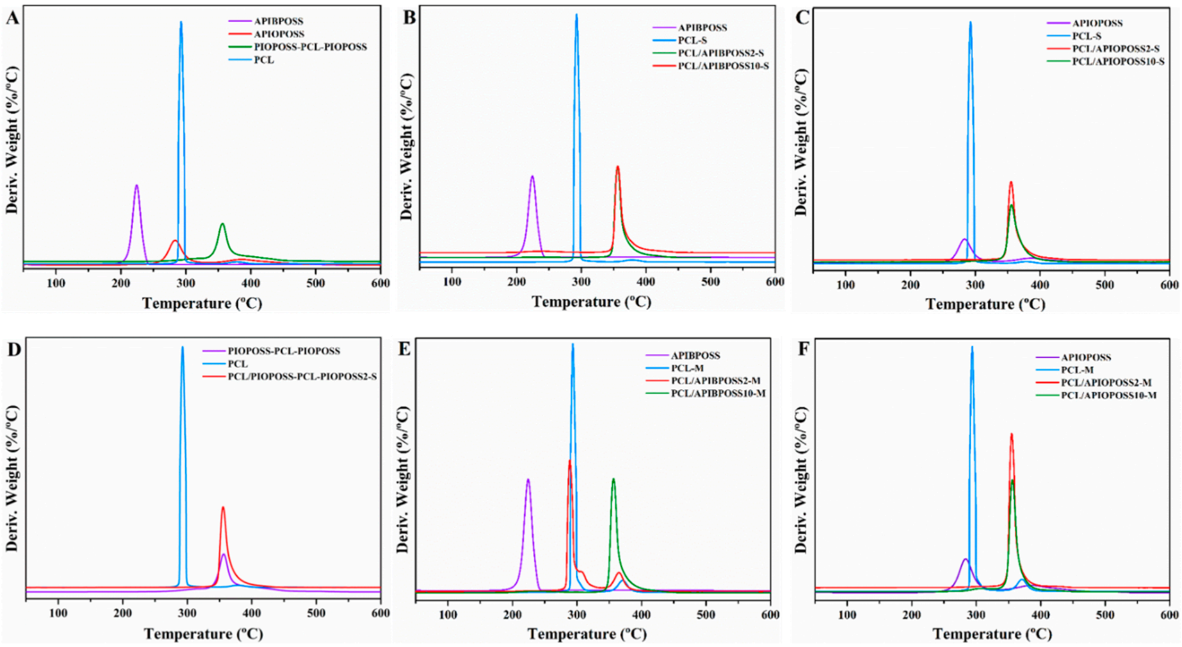

Figure S2. DTG curves of: (A) nanofillers and neat PCL, (B) (C) and (D) solution blended nanocomposites, (E) and (F) melt mixed nanocomposites in N_2 atmosphere.

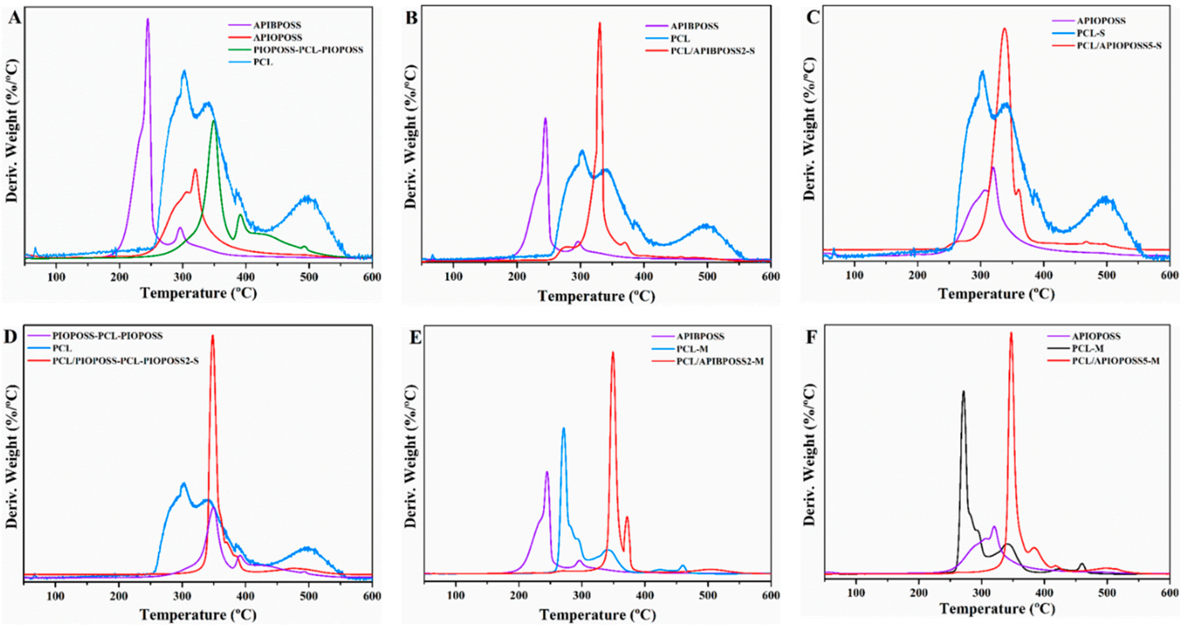

Figure S3. DTG curves of: (A) nanofillers and neat PCL, (B) (C) and (D) solution blended nanocomposites, (E) and (F) melt mixed nanocomposites in O_2 atmosphere.

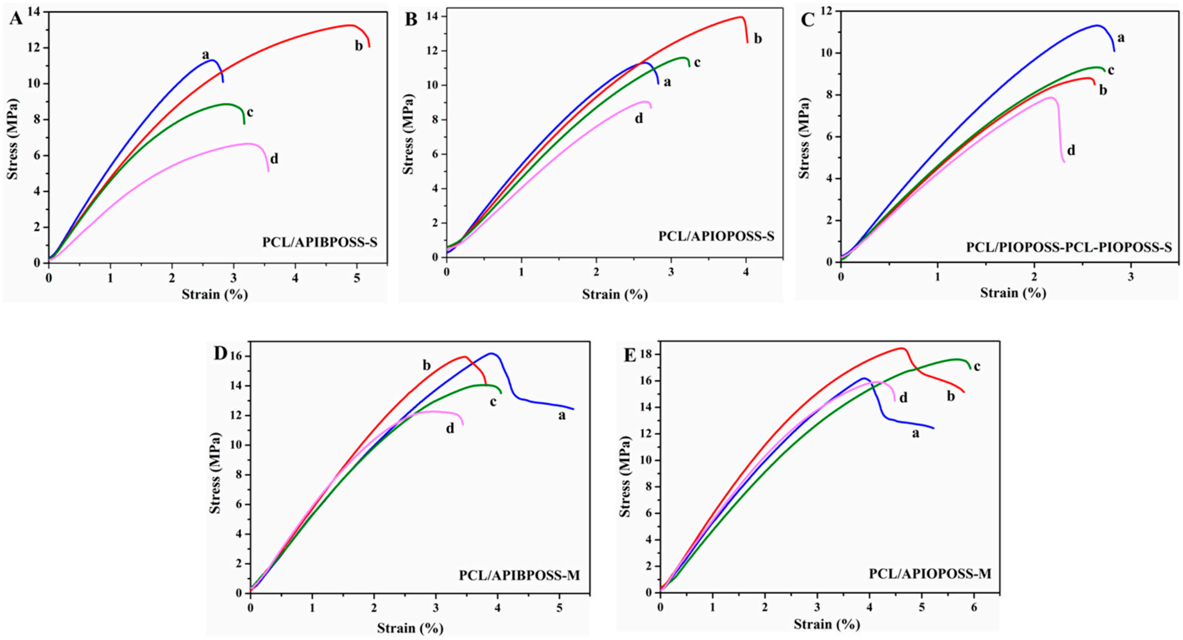

Figure S4. Stress-strain curves A, B and C: solution blended nanocomposites; D and E: melt mixed nanocomposites; (a) PCL, (b) 2 wt% POSS, (c) 5 wt% POSS, and (d) 10 wt% POSS.

Table S2. Contact angles of neat PCL, PIOPOSS-PCL-PIOPOSS and PCL/POSS nanocomposites.

Sample	WCA
PCL	70.0±0.7
PIOPOSS-PCL-PIOPOSS	97.1±0.7
PCL/APIBPOSS 2	103.5±0.5
PCL/APIBPOSS 5	105.5±1.1
PCL/APIBPOSS 10	102.9±1.1
PCL/APIOPOSS 2	96.9±1.1
PLLA/APIOPOSS 5	96.7±1.2
PLLA/APIOPOSS 10	97.6±1.2
PCL/PIOPOSS-PCL-PIOPOSS 2	95.4±0.7
PCL/PIOPOSS-PCL-PIOPOSS 5	95.7±0.6
PCL/PIOPOSS-PCL-PIOPOSS 10	95.0±1.7
